# Bipolar radiofrequency catheter ablation of incessant ventricular tachycardia originating from anterolateral papillary muscle

**DOI:** 10.1016/j.hrcr.2024.08.009

**Published:** 2024-08-13

**Authors:** Vera Maslova, Thomas Demming, Fabian Moser, Romina Krueger, Derk Frank, Evgeny Lian

**Affiliations:** ∗ Department of Internal Medicine III, Cardiology and Angiology, University Hospital Schleswig-Holstein, Kiel, Germany; † Abbott Medical, Minneapolis, Minnesota

**Keywords:** Catheter ablation, Ventricular tachycardia, Bipolar ablation, Papillary muscle, Case report

**Key Teaching Points**

• Bipolar ablation could be an alternative treatment option for ventricular tachycardia originating from the papillary muscle when an intramural substrate is suspected, and other ablation strategies have failed.
• The correlation of CT findings, electrograms, and activation maps represents the intramural substrate and emphasize the need of deeper ablation, using the bipolar configuration.
• Catheter ablation of PVC arising from papillary muscles using point-by-point mapping without use of intracardiac echocardiography is feasible.

## Introduction

Radiofrequency catheter ablation (RFCA) of the ventricular arrhythmias, originating from the papillary muscle (PM) can be challenging because of its deep origin, complex PM anatomy, and poor catheter stability, which leads to unsatisfactory acute (88.1%) and long-term (69.2%) success rates.^1^ Unipolar RFCA owes a limited ability to create transmural lesions, while bipolar RFCA allows deeper lesion formation.^2^ Therefore, bipolar RFCA (Bi-RFCA) has been reported to be effective in cases of the deep intramural location of the arrhythmogenic substrate.^3^, ^4^, ^5^, ^6^ Successful cases of the Bi-RFCA of the premature ventricular contractions (PVC) originating from the PM have been reported.^7^^,^^8^ This is the first case reporting Bi-RFCA of ventricular tachycardia (VT) originating from the PM.

## Case report

**Figure 1:**
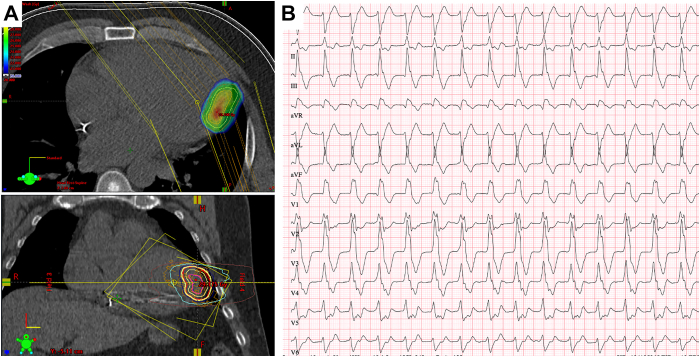
**A:** First unipolar ablation procedure, right anterior oblique view. **B:** Radioablation treatment plan images, axial (above) and frontal (below) views. **C:** Electrocardiogram of the ventricular tachycardia on the admission (speed = 25 mm/s).

A 60-year-old man presented to the emergency department with palpitations and dyspnea. The patient had a history of dilated cardiomyopathy with now-normal left ventricular ejection fraction under the heart failure medication and a single-chamber implantable cardioverter-defibrillator (ICD), which had been implanted for secondary prevention. Previously performed computed tomography of the heart with late enhancement showed intramural fibrosis in the left ventricular lateral wall below the ALPM (Supplemental Video 1). One year ago, the patient had already undergone unipolar RFCA of the anterolateral PM (ALPM) VT (Figure 1A). Then, owing to VT recurrence, another unipolar RFCA attempt was anticipated, but because of the absence of substrate and pathologic signals on the endocardial mapping, a decision against it was made, and radioablation of the same area was performed (Figure 1B). However, the patient had recurrent multiple VT Episodes, requiring ICD therapy during the last 5 months after the radioablation.

ECG on the admission showed a slow VT with a heart rate of 112 bpm, inferior axis, and right bundle branch block morphology (Figure 1C), suggesting the exit of the VT at the ALPM. The patient was hemodynamically stable. At the time of recurrence, the patient had antiarrhythmic medication with propranolol in maximum dose and flecainide 100 mg twice daily as an off-label therapy. Multiple attempts of burst pacing through the ICD led to the termination of the VT with further spontaneous reinduction despite intravenous amiodarone infusion prompting another RFCA.

**Figure 2:**
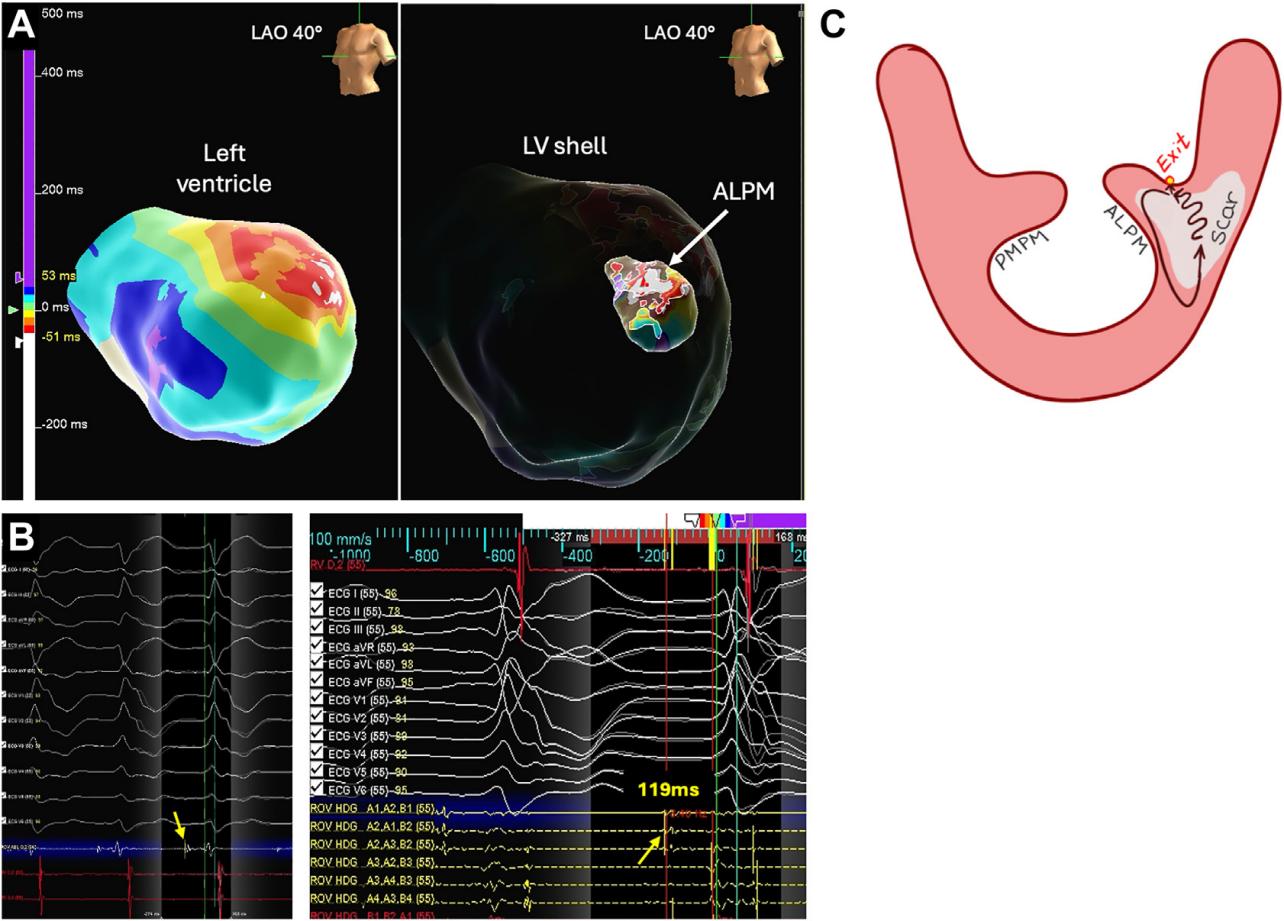
**A:** Left: activation map of the LV endocardium with the white color, representing the earliest activation site in the middle of the ALPM (white arrow). Right: Activation map of ALPM inside the LV shell. **B:** Diastolic potential recorded at the body of the ALPM by ablation catheter (on the left, yellow arrow) and by the multielectrode catheter (on the right, red arrow). **C:** Suggested VT mechanism. ALPM = anterolateral papillary muscle; LV = left ventricle; VT = ventricular tachycardia.

**Figure 3:**
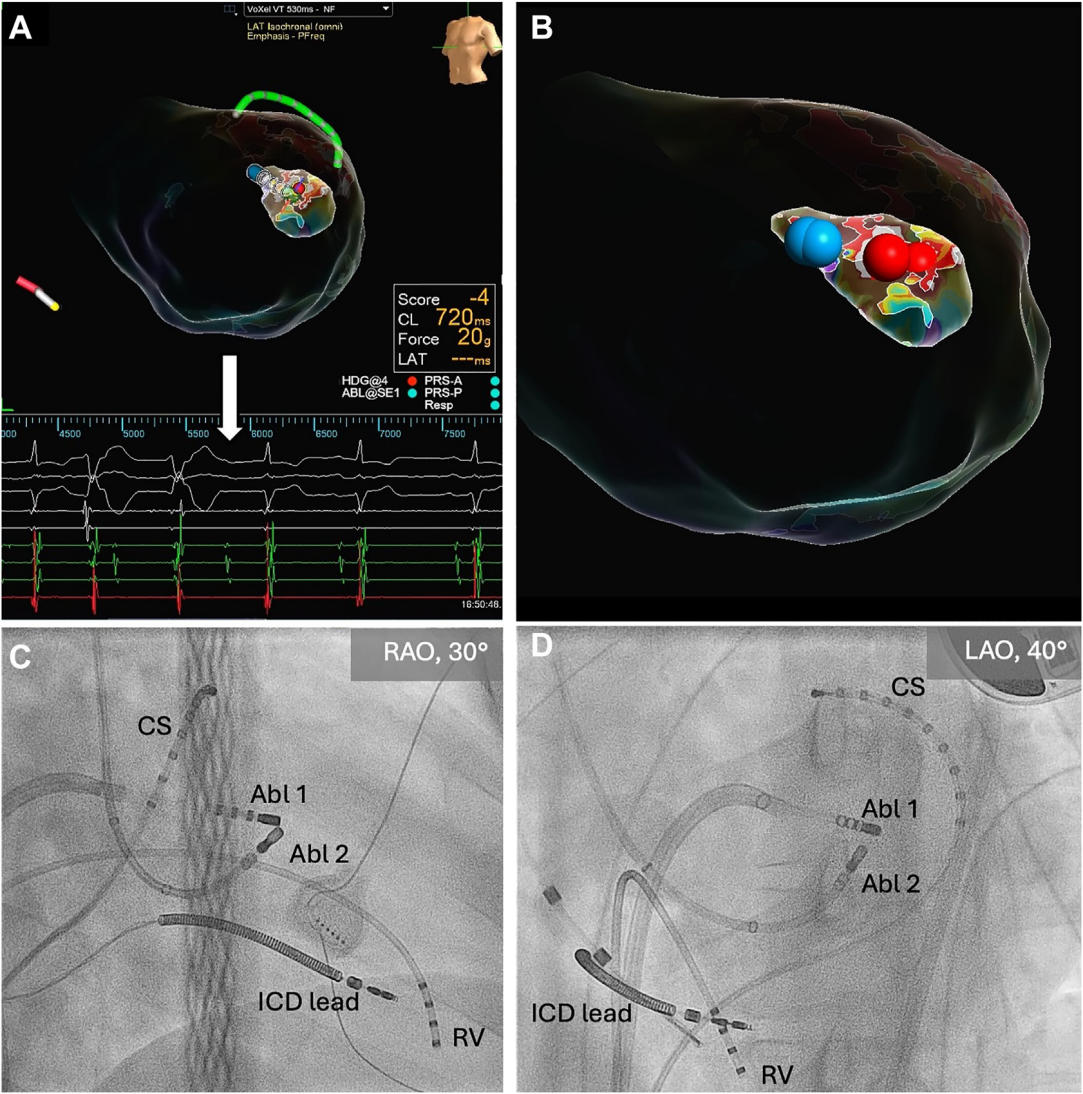
**A:** Termination of the ventricular tachycardia (white arrow) during the unipolar ablation. **B:** Ablation points: blue, bipolar; red, unipolar. **C and D:** Catheter positions for the bipolar ablation of the papillary muscle. Abl 1 = ablation catheter transseptal; Abl 2 = ablation catheter transaortic; CS = coronary sinus, RV = right ventricle.

The procedure was performed using conscious sedation. The electroanatomic map of the left ventricle (LV) and PM was obtained using a 3-dimentional mapping system (EnSite^TM^, Abbott, Minneapolis, MN) with a multielectrode mapping catheter (Advisor HD Grid Catheter, Abbott) via transseptal approach with steerable sheath (Agilis^TM^, Abbott). The endocardial voltage map showed no relevant low-voltage areas. The activation map of the LV during the VT revealed a focal pattern with the earliest activation site in the anterolateral wall of the LV corresponding to the base of the ALPM (Figure 2A). At the site of the body of ALPM, sharp diastolic potentials were recorded, with the earliest preceding the surface QRS by 119 ms (Figure 2B). As an underlying VT mechanism, we suspected an intramural reentry in the scar area with an intramural VT isthmus and an exit site at the base of ALPM (Figure 2C), which caused the focal pattern on the activation map. The entrainment was not performed. The reconstruction of the ALPM was performed manually by acquiring the points with the activation time preceding the QRS onset and force value more than 2 g, while intracardiac echocardiography was not available. Initially, unipolar ablation with 50 W at the site of the earliest diastolic potential was performed with successful termination of the VT (Figure 3A). However, the VT could still be induced with pacing maneuvers, suggesting a deep intramural substrate, unreachable even for high-power unipolar RFCA. That prompted the Bi-RFCA of the ALPM using two irrigated ablation catheters: a TactiFlex ablation catheter (Abbott), as an “active” catheter advanced transeptally, and a Thermocool SF (Biosense Webster, Diamond Bar, CA), as a “return” catheter advanced transaortic and connected to the generator (Ampere RF Ablation Generator^TM^, St. Jude Medical, St. Paul, MN) via “Dr. Futyma” bipolar ablation adapter (CorSystem). The irrigation of both catheters was performed with normal saline (0.9%). The catheters were placed on the opposite sites of the ALPM (Figure 3B) with an average force on the active catheter of 18 g. The baseline bipolar impedance was 144 Ω. Two bipolar lesions were delivered during sinus rhythm with 30 W of power and a maximum temperature of 44°C, achieving an average impedance drop of 15%, which resulted in non-inducibility of VT native and under the isoproterenol infusion. Total RF time was 283 seconds, bipolar RF time 98 seconds, and unipolar RF time 185 seconds. The procedure lasted 119 minutes. No complications, including steam pop, were observed. In postprocedural echocardiography, there were no signs of mitral regurgitation and no signs of papillary muscle rupture. Therapy with propranolol was continued. During the 2-month follow-up, the patient was free from the VT.

## Discussion

This is the first report of bipolar ablation of the VT with the involvement of the PM. Ventricular arrhythmias from PM are rare, are mostly idiopathic, and originate mainly from the posterior PM.^9^ In our patient, the VT was probably scar-related, with the exit site on the base of the ALPM.

Because of the two previously failed ablation attempts, the presence of the intramural substrate in the anterolateral wall below the ALMP, and VT inducibility after the unipolar ablation, an alternative approach with Bi-RFCA was used to improve the outcome in this challenging scenario.

For the Bi-RFCA, two ablation catheters create an ablation circuit, which increases the radiofrequency current delivered to the substrate and facilitates deeper lesion formation.^6^ In our case, a comprehensive activation mapping was possible, thanks to the good hemodynamical tolerance of the VT. Activation mapping revealed the earliest activation site at the base of the ALPM. The potentials, recorded at the body of the ALPM and preceded the QRS onset by 119 ms, could be interpreted as a late diastolic part of the reentry circuit with the early diastolic part located intramurally. The presence of intramural fibrosis in this area, stable CL, and induction through the pacing maneuvers support the reentry mechanism. However, the ectopic mechanism with an origin in the body of ALPM and very slow propagation to the base of the PM owing to previous ablation procedures cannot be excluded.

The usage of small-tip ablation catheters could be associated with large baseline impedance values and higher temperature rise.^10^ Arai and colleagues^8^ reported high impedance values >200 Ω during the Bi-RFCA of PVC from ALPM, which prompted the usage of a large-tip return catheter. In our case, the baseline impedance was 144 Ω and did not affect the ability to deliver the RF current. The temperature rose within the normal range, and no steam pops were observed.

Possible periprocedural complications of Bi-RFCA of the PM may include a rupture of the PM or chordal apparatus, which was not observed in our case. Our limitation in this case is that the intraprocedural visualization with intracardiac echocardiography was not available. Moreover, as the entrainment maneuver has not been performed and we failed to demonstrate the early diastolic part of the reentry circuit, the reentry mechanism of the VT cannot be definite. That is why the reconstruction of the ALPM was performed manually by including only the points with the activation timing preceding QRS onset.

## Conclusion

Bipolar ablation could be an alternative treatment option for ventricular tachycardia originating from the papillary muscle when an intramural substrate is suspected, and other ablation strategies have failed.

## Disclosures

The authors have no conflicts to disclose.
